# Schistosomes in the Lung: Immunobiology and Opportunity

**DOI:** 10.3389/fimmu.2021.635513

**Published:** 2021-04-19

**Authors:** Emma L. Houlder, Alice H. Costain, Peter C. Cook, Andrew S. MacDonald

**Affiliations:** ^1^Lydia Becker Institute of Immunology and Inflammation, Manchester Collaborative Centre for Inflammation Research, Faculty of Biology, Medicine and Health, Manchester Academic Health Science Centre, University of Manchester, Manchester, United Kingdom; ^2^MRC Centre for Medical Mycology, University of Exeter, Exeter, United Kingdom

**Keywords:** schistosomiaisis, lung, helminth, acute, pulmonary, Katayama syndrome

## Abstract

Schistosome infection is a major cause of global morbidity, particularly in sub-Saharan Africa. However, there is no effective vaccine for this major neglected tropical disease, and re-infection routinely occurs after chemotherapeutic treatment. Following invasion through the skin, larval schistosomula enter the circulatory system and migrate through the lung before maturing to adulthood in the mesenteric or urogenital vasculature. Eggs released from adult worms can become trapped in various tissues, with resultant inflammatory responses leading to hepato-splenic, intestinal, or urogenital disease – processes that have been extensively studied in recent years. In contrast, although lung pathology can occur in both the acute and chronic phases of schistosomiasis, the mechanisms underlying pulmonary disease are particularly poorly understood. In chronic infection, egg-mediated fibrosis and vascular destruction can lead to the formation of portosystemic shunts through which eggs can embolise to the lungs, where they can trigger granulomatous disease. Acute schistosomiasis, or Katayama syndrome, which is primarily evident in non-endemic individuals, occurs during pulmonary larval migration, maturation, and initial egg-production, often involving fever and a cough with an accompanying immune cell infiltrate into the lung. Importantly, lung migrating larvae are not just a cause of inflammation and pathology but are a key target for future vaccine design. However, vaccine efforts are hindered by a limited understanding of what constitutes a protective immune response to larvae. In this review, we explore the current understanding of pulmonary immune responses and inflammatory pathology in schistosomiasis, highlighting important unanswered questions and areas for future research.

## Introduction

Schistosomiasis is a neglected tropical disease, with over 200 million people infected by trematodes of the *Schistosoma* genus – *S. mansoni*, *S. haematobium* and *S. japonicum* (1). Infection occurs when individuals come into contact with water containing cercariae, which infect humans *via* the skin (2–4). After skin penetration, cercariae transform into larval schistosomula which enter the bloodstream, passing through the lungs before maturing in the hepatic portal veins, where males and females pair and move to either the mesenteric (*S. mansoni* and *S. japonicum*) or urogenital (*S. haematobium*) vasculature to lay eggs (5). Schistosomiasis can be categorised into acute and chronic phases. The acute phase encompasses larval migration, maturation and initial egg production (6). Symptoms of the acute phase, most commonly observed in travellers to endemic regions, include cercarial dermatitis in the days following infection, and Katayama syndrome, characterised by fever, cough and myalgia, from week 2 post infection (7). The chronic stage of schistosomiasis has been proposed to begin, somewhat arbitrarily, at 12 weeks post infection, with egg-deposition driving symptoms in multiple organs including the liver, bladder, and lung (8). Approximately 60% of endemically infected individuals experience schistosomiasis associated morbidity, predominantly anaemia and malnutrition, with 10% severely ill (5, 9, 10). Upon closer inspection, severely ill individuals often harbour high intensity infections and appear immunologically inept at regulating the persistent, egg-driven pathology (11).

**Figure 1:**
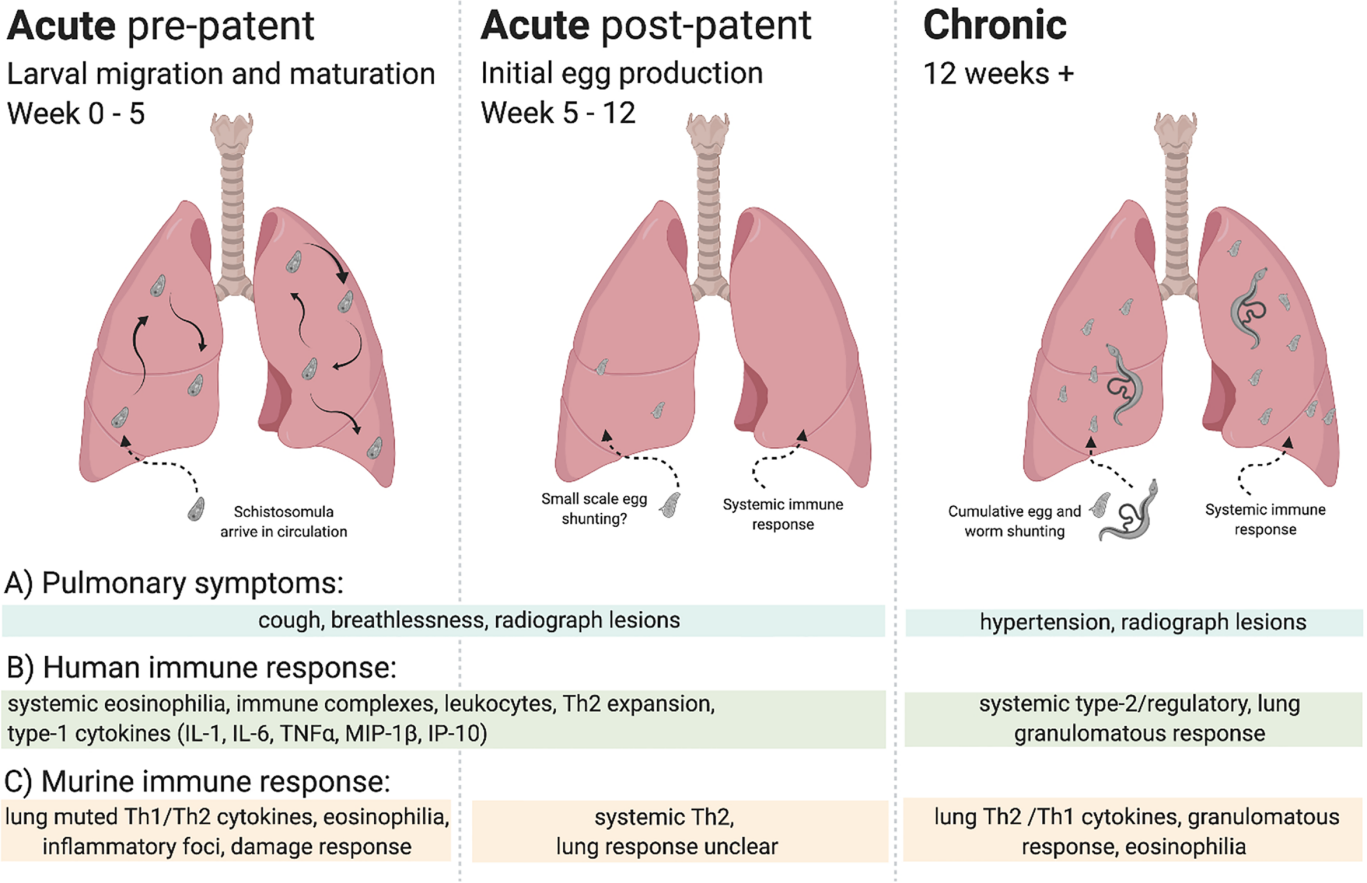
Overview of lung immune responses during *S. mansoni* infection. Lung immune responses differ depending on the life cycle stage of the parasite, broadly characterised into acute (pre-patent and post-patent) and chronic phases. **(A)** Pulmonary symptoms vary, with cough and breathlessness during the acute phase in response to larval migration, maturation, and initial egg production. In chronic disease pulmonary hypertension is the key pulmonary pathology. Lesions may be observed in lung radiographs in all stages, although with differing aetiology in response to migrating larvae, worms, or eggs. **(B)** Human immune responses can be split into acute and chronic infection, often assayed systemically. Broadly, an early eosinophilic response with mixed type-1/type-2 cytokines makes way for a type-2/regulatory response to schistosome eggs, with tissue granulomas. **(C)** Murine immune responses can be more accurately demarcated into stage-specific responses, with a muted type-1/type-2 response to lung migrating larvae making way to a systemically driven dominant type-2 response upon egg production. In response to pulmonary egg deposition, chronic responses in the lung display type-1/type-2 cytokine production and granulomatous inflammation.

Although lung symptoms can occur in both acute and chronic schistosomiasis, as shown in **Figure 1**, the aetiology of pulmonary pathology in acute schistosomiasis is not well understood (12). Pulmonary symptoms – shortness of breath, wheezing and dry cough (13) – can begin before larvae develop to adulthood and patency (egg production), as early as 2 weeks post infection, and therefore may be attributable to immune responses to lung migrating schistosomula (14, 15). Alternatively, at the post-patent acute stage these same symptoms may be caused by a systemic immune response to worms or eggs in the mesenteric or urogenital vasculature (13). In these cases, eosinophils, induced in response to parasite antigens, may be sequestered in the lungs, observable as lesions on lung radiographs (15, 16). In chronic schistosomiasis, potentially years post infection, pulmonary symptoms are better understood. Eggs, or even adult worms, were identified in the lungs of patients with chronic disease over 80 years ago (17). Further, pulmonary egg deposition due to advanced hepatosplenic disease can lead to potentially fatal pulmonary hypertension (17–23).

The immune response to schistosomiasis changes over the course of infection, dictated both by developmental changes in the parasite and tissue location, as well as host immune adaption to long-term infection (24). A classic example of this would be the onset of patency which, in murine models, provokes a dramatic increase in Th2 (IL-4 and IL-5) cytokine production (25). Despite ongoing infection, schistosome antigen responsiveness (26), and Th2 cytokine production is thought to peak around week 8-10 post infection, then reduce (27), with a concurrent increase in regulatory T and B cells, and T cell hyporesponsiveness (28–31). This shift to an increasingly regulatory phenotype from about week 12 post infection is thought to underlie the transition between acute and chronic schistosomiasis (27). Therefore, where possible, we will use ‘pre-patent acute’ (0 to 5 weeks post infection), ‘post-patent acute’ (5 to 12 weeks post infection), and ‘chronic’ (>12 weeks post infection) terminology to refer to the different phases of infection in this review.

Murine studies have provided valuable insight into the pulmonary immunological response to acute and chronic schistosomiasis. During the pre-patent lung migrating stage, there is some evidence that a pulmonary Th2 response is initiated (32), with eosinophilic pulmonary infiltrates (14), and a low-level systemic Th1 response (25, 32). In the patent stage of infection, pulmonary inflammation is thought to reflect the exaggerated systemic Th2 responses driven by schistosome eggs in organs such as the liver (25) until eggs translocate to the lungs *via* portal shunts (peaking around week 17), leading to a similarly Th2 dominated granulomatous response directly in the lung tissue (33, 34). Since schistosome larvae migrate through the lungs early in infection (35), a deeper mechanistic understanding of pulmonary immune responses will be critical for development of vaccines (36, 37), as well as treatments against schistosomiasis associated pulmonary hypertension (38).

### Schistosomula Migration

The precise timing of schistosomula lung migration in humans is not well understood. In a human model of *S. mansoni* skin infection, transformed cercariae were seen in the epidermis 4 hours after exposure, remaining there for over 24 hours, before moving through the dermis and hypodermis to the blood vessels by 48-72 hours post infection (39). The timing of human skin migration by *S. haematobium* is comparable to *S. mansoni*, entering the dermis at around 48 hours post infection. In comparison, *S. japonicum* human skin migration occurs much faster, with the majority of schistosomula reaching the dermis by 9 hours post infection (39). Schistosomula lung migration, larval maturation and pairing then occurs over a number of weeks, with initial egg production evident in faeces or urine by 5-7 weeks post infection in humans (6). Understanding what occurs in these weeks can be informed by primate models, in which tracking studies are possible. In baboon primary infection, *S. mansoni* larvae remain in the skin at day 2 post infection, moving to the lungs by day 5, with the majority having left the lungs and detectable in the liver by day 9 (40). To our knowledge, primate tracking studies of larval migration have not been performed for *S. japonicum* or *S. haematobium* infection.

Murine models have also been used to study larval migration, with skin migration in mice following a similar timeline to humans. In primary infection studies, *S. mansoni* penetrates the epidermis in minutes (2, 41, 42), but then takes approximately 72 hours to migrate, first to sebaceous glands or epidermal ‘migration channels’, then to the connective tissue of the dermis and hypodermis, before penetration of blood vessels as confirmed by histological analysis of skin sections (43). Schistosomula are then swept in the blood system to the heart, where they travel into the pulmonary circulation (44). Utilising histological analysis as well as autoradiography of infected mice, *S. mansoni* has been found in the lungs from day 4, peaking at day 10-12, and steadily declining until approximately day 20, when they start to be detectable in the mesenteric vessels and liver (43, 45). Larvae may make multiple circuits of the vasculature before reaching the portal system (35). Timing in other species differs, with *S. japonicum* arriving in the lungs earlier, peaking at day 3 (46), and *S. haematobium* migrating more slowly, arriving in the lungs at day 7 (47).

### Symptoms and Immunology of Acute Schistosomiasis

Acute schistosomiasis, or Katayama syndrome, can occur any time between 2 to 12 weeks post infection, which encompasses larval migration and initial egg deposition (7). Katayama syndrome has been reported to occur in the majority (over 50%) of travellers infected with *Schistosoma* spp., but is rare in endemic individuals in schistosomiasis affected countries (15, 48). One explanation for this striking disparity is that endemic locals are exposed to schistosome antigens and/or antibodies in utero, causing a subsequent modulated immune response (49).

The predominant pulmonary symptoms observed in Katayama syndrome are cough and lesions observed on lung radiographs (15, 23). Radiographically observed lung lesions have the capacity to self-resolve, or can be treated with the anthelmintic praziquantel (50). These lesions, caused by immune cell infiltrates, take on a variety of different forms, from a diffuse interstitial pattern to nodular ‘ground glass’ presentations (12). The variety in lesion presentation has been proposed to be related to the stage of the disease (12). For instance, early, diffuse infiltrates may result from a systemic hypersensitivity response to migrating and maturing larvae, with morphological features of eosinophilic pneumonia, as seen in other parasitic lung infections (15, 51). These lesions have been observed in patients as early as 4 weeks post infection, and can occur prior to eggs being detectable in stool (15, 52). Nodular lesions have been shown to occur in response to clusters of schistosome eggs, with histological analysis of lung biopsies revealing eosinophilic granulomas surrounding these eggs (53). However, recent studies have supported the idea that egg deposition is not required for Katayama syndrome (54, 55).

In travellers infected with an unusual *S. haematobium/S. mattheei* hybrid, shown to be infertile, symptoms of acute schistosomiasis developed 16-41 days post exposure, with fever, muscle ache and cough the most common symptoms (present in 50-69% of patients). Notably, schistosome eggs were not detected in stool or urine (via PCR or microscopy), although parasite infection was confirmed by PCR on serum (55). Katayama syndrome also occurred in a pioneering study of human experimental schistosome infection with male parasites alone (preventing egg production) (54), although only 1 out of 17 (6%) of the volunteers experienced pulmonary symptoms (cough) (56). These cases are supported by multiple reports documenting acute schistosomiasis as early as 2 weeks post infection, when egg production has not yet begun (57, 58). Chest radiographs were not performed in these studies, and so the presence of lung lesions could not be determined.

Post-patent acute schistosomiasis displays characteristic immunological features, with the majority of patients presenting with significantly increased blood eosinophilia, peaking in the initial stages of egg production, at 4-6 weeks post infection (57, 59). Eosinophils are known to produce a number of substances, including prostaglandin E_2_ and substance P, that may induce coughing, potentially explaining this symptom (60). Notably, in human infection with single sex parasites, peripheral blood eosinophilia was also observed in 65% of volunteers, peaking between weeks 4-8, suggesting adult worms are sufficient to drive eosinophilia even in the absence of egg production (54, 56). Observational studies have also shown increased total blood leukocyte levels towards the end of the acute phase (8-12 weeks post exposure) (59). In addition to this, immune complexes (formed by multiple antibodies binding antigen) have been observed from 8 weeks post exposure in 55-93% of people with acute schistosomiasis, but are absent in non-infected controls (61). *S. mansoni* egg antigens may be critical for these immune complexes, as supported by work showing immune complex levels are correlated with stool egg counts, and first appear in murine models at day 35 post infection, when initial egg production is expected (62, 63). Circulating immune complexes during post-patent acute schistosomiasis have been shown to be positively associated with the pulmonary symptoms of cough, dyspnoea and interstitial infiltrates observed in chest radiographs (64), suggesting a potential causative link. A strong correlation was observed between *S. mansoni* faecal egg counts and severity of symptoms of acute schistosomiasis, suggesting that infection load is a key influencer of the condition (57).

Immunologically, acute human schistosomiasis has traditionally been proposed to be a type-1 dominated response (24). Key evidence for this is a study showing secretion of the type-1 associated cytokines IL-1, IL-6 and TNFα elevated in unstimulated PBMCs from patients with post-patent acute schistosomiasis (4-9 weeks post exposure), in comparison to patients with chronic disease (64). However, Th2 responses were not thoroughly investigated in this paper, with only one Th2 cytokine (IL-5) assessed, detectable in PBMCs from acute schistosomiasis patients stimulated with schistosome egg antigen (SEA) or schistosome worm antigen (SWA). Without healthy controls reported, it is impossible to know if the levels of IL-5 were increased compared to baseline (64). Further studies, with a broader range of Th2 cytokines, are required before the immunological bias of acute human schistosomiasis can be confidently stated.

Evidence for a mixed pro-inflammatory/type-1 and Th2 immune response in pre-patent acute schistosomiasis comes from experimental human infection with single sex parasites. In this study, serum levels of inflammatory and type-1 associated chemokines macrophage inflammatory protein 1β (MIP-1β) and interferon gamma induced protein 10 (IP-10) were increased at week 4 post infection, with an increase in MIP-1β correlated to symptoms of acute schistosomiasis (56). However, this study also provided evidence for the importance of the Th2 response in acute schistosomiasis, with antigen-specific Th2 (IL-4, IL-5 and IL-13), but not Th1 (IFNγ), CD4^+^ T cells at week 4 post infection, significantly correlated to acute symptoms (56). This prominence of the Th2 response is consistent with multiple previous reports of eosinophilia during pre-patent acute human schistosomiasis, with eosinophils often regarded as a type-2 effector cell (57, 59, 65).

Much less is known about immune responses to migrating and maturing larvae at 0-3 weeks post infection, perhaps due to the difficulty of identifying individuals so soon after exposure. Symptomatic acute schistosomiasis can first occur from 2 weeks post exposure (66), suggesting immune responses may begin at this early stage. Some insight into the human response to schistosomula may be gained from a study of infected endemic individuals where PBMC responses were measured to recombinant schistosomula antigens. These antigens provoked type-1 and proinflammatory (including IFNγ, IL-1β, TNFα), as well regulatory (IL-10, IL-1Ra) cytokine responses (67). Although informative, it is not clear whether the response to these antigens, chosen due to their identification as vaccine targets, is representative of the overall response to migrating larvae in natural infection. Moreover, responses to these antigens may be altered by their continued expression in adult stage worms (68). Further studies of immune responses during human pre-patent acute schistosomiasis are required to better understand this phase of the disease.

### Immunology of Acute Schistosomiasis in Murine Models

Although most murine studies have focused on primary infection, repeat or “trickle” infections beginning in childhood are more likely to represent human schistosome interactions in endemic populations (69). However, one issue with studying repeat infections over a long time period in mouse models is that egg-induced responses can modify the hepatic vasculature, impairing the ability of schistosomula from subsequent infections to lodge and mature in the liver. This means that parasites can instead end up in the lung vasculature, where they are eliminated, presumably mediated by the immune response (70). This “leaky liver” model of resistance to reinfection is not thought to be replicating the state observed in chronic human infections, which has perhaps discouraged the use of murine infection beyond primary infections or repeated infections over a shorter time period (71). In addition, there has been much debate on how to best mimic “natural” infectious doses in mouse models. Controlled infection studies of non-endemic individuals showed that infection with 10-30 cercariae was sufficient to lead to diagnosable infection in all volunteers and infection intensity levels comparable to those seen in a low burden endemic setting (56, 72). This compares favourably to infectious doses used in numerous murine studies, which tend to range from 20 – 80 cercariae (24). However, more caution is likely needed when interpreting reports that have used very high doses of parasites (up to 500), which may partially explain some previously highlighted immune response disparities between humans and murine models (73). Importantly, it should be emphasised that few reports (regardless of infection dose) have studied lung-stage immunity closely and that additional research is needed to understand how infectious dose alters early immune events in the lung. These considerations must therefore be met with pragmatism: “pushing the system” with an increased primary infectious dose may be required to observe, and therefore mechanistically interrogate, the fundamentals of the immune response to schistosome larvae.

One distinct advantage of murine models is the ability to dissect *in vivo* responses to the parasite at defined timepoints post infection. However, in comparison to the wide literature that exists addressing liver, spleen, and mesenteric lymph node immune responses during murine schistosomiasis, relatively few studies have investigated the lung stage of infection. Assessment of thoracic (lung draining) lymph node T cell responses during schistosomula lung migration revealed a significant increase in Th2 cell IL-4 expression, but not Th1 cell IFNγ, at days 14 and 21 post 70 cercariae infection (32). It has also been suggested that promotion of Th2 responses by larval antigens may prime the immune response in advance of egg deposition, aiding both host and parasite survival (74). Additional studies utilising state of the art techniques to expand this limited understanding of pulmonary inflammation during schistosome migration are sorely needed.

Older histological studies have, in high dose (450 cercariae) infections, observed inflammatory foci consisting of neutrophils, eosinophils, macrophages and lymphocytes around lung migrating *S. mansoni* larvae at days 7-31 post infection (14). Notably these foci were not seen surrounding intravascular parasites, but only those that had entered the alveolar space, supporting a model in which “failed” intra-alveolar larvae, which have burst out of the narrow lung capillaries, may be the main drivers of pulmonary inflammation against migrating parasites (14, 35).

Similar results were observed during high dose (400-500 cercariae) murine infection with *S. japonicum*, with leukocyte, macrophage and eosinophil infiltration observed in murine lungs from 3-5 days post infection (75). In addition to this, histopathological changes were observed in the lung parenchyma and vessels, with vasculitis, haemorrhage and parenchymal involvement from 5 days post infection (75). No direct association between intravascular larvae and immune infiltration was noted, potentially suggesting a non-specific damage response, a hypothesis supported by transcriptional data from lung tissue, in which upregulation of wound healing genes was observed (75). In addition to this, lung inflammatory gene signatures increased post infection, including chemokines CCL7, CCL17 and CCL2, as well as the gene *Retnla* (Resistin Like Molecule alpha, RELMα), which is commonly upregulated during type-2 inflammation (75). Further work is required to understand which pulmonary cell types are responsible for the upregulation of these mediators, and their potential functional roles, with our current understanding of innate cells particularly lacking in this context.

Finally, systemic immune parameters measured during murine lung migration have also been used to proxy pulmonary immune responses to schistosomula. 2-4 weeks post infection with 40 cercariae, *S. mansoni* antigen stimulated splenocytes show increased expression of IFNγ, as well as the Th2 cytokines IL-5 and IL-4, when compared to uninfected controls (25, 74). Looking slightly earlier, at day 9 of 100-150 cercariae *S. mansoni* infection, an increase in plasma levels of the Th17 associated cytokine IL-17, in addition to the Th2 cytokine IL-4 and the Th1 promoting cytokine IL-12p70, was observed (76). Although of reduced relevance to pulmonary responses, systemic measurements are more easily comparable with human studies, in which blood is often the most accessible sample.

### Immune Evasion by Lung Migrating Schistosomula

When considering how schistosomula may evade the immune response while migrating through the lung, the anatomical structures of the airways and vasculature must be considered. It is unclear whether pulmonary migration is beneficial for the parasite, or instead an inconvenient obstacle to bypass. Both tissue resident and circulating immune cells patrol the lower airways and lung capillaries. However, in order for the lungs to constantly carry out their vital physiological role of respiration, immune responses in the lower airways are tightly controlled to maintain homeostasis (77). A wide array of pulmonary innate defences against common airborne pathogens are therefore instead concentrated on the upper airways (78). The regulatory bias of immunity in the lower airways may provide a relatively protected environment for larval development, proposed to explain why larval lung migration is a feature of multiple other helminth life cycles (78). Another important consideration is the constant movement of larvae within the vasculature, which may make localising a focal immune response by patrolling immune cells, such as neutrophils, more difficult (79). Thus, their intravascular niche, in combination with motility and relatively rare ‘rupturing’ into the airways, may together help limit inflammation around the migrating schistosome larvae.

On the other hand, the time taken for schistosomula to transit through murine lung capillary beds, 30-35 hours in comparison to 6-16 hours in systemic and intestinal capillary beds, suggests the lung may present a unique migratory obstacle (80). After arriving in the lung, 18μm wide schistosomula (81) must narrow and elongate to bypass thin walled and 6μm wide lung capillaries (82). Schistosomula must remain entirely intravascular for successful migration, as rupturing into the alveoli risks them being swept up the airways, swallowed and digested (83). Moreover, damage caused by rupturing into the alveolar space may invite immune attack (14).

Schistosomes employ numerous mechanisms in order to evade immune attack, as detailed below, and recently reviewed (84). Lung stage larvae have been shown to coat themselves in host antigens, including blood group antigens and major histocompatibility (MHC) proteins, in order to block binding of host anti-schistosomula antibodies (85–87). In adult worms this has been shown to be an active process, with coating of human low density lipoprotein (LDL) dependent upon specific binding to parasite receptors (88). However, unlike adult worms, when this defence is overcome *via* addition of anti-host antibody to lung stage schistosomula *in vitro*, larvae are still resistant to immune killing (85). This could be explained by an intrinsic resistance of lung stage schistosomula to antibody dependent killing, potentially due to ongoing breakdown and biosynthesis of schistosomula membrane sphingomyelin allowing access of small molecules, but not antibodies, to the parasite surface (89, 90). Intrinsic properties of the schistosomula outer coat (tegument) may allow it to resist immune attack, as they rapidly acquire a protective outer lipid bilayer, referred to as the membranocalyx, during development from cercariae (91–93). In addition, schistosomula are able to evade complement killing *via* the acquisition of host decay accelerating factor (DAF), which prevents assembly of complement convertases critical for both the alternative and classical pathway of complement activation (94).

As well as acquisition of host products, the parasites themselves produce molecules that may contribute to immune evasion. As schistosomula predominantly stay intravascular, they have been found to suppress immune activation of lung endothelial cells (95). Specifically, schistosomula can interfere with the NF-κB pathway in endothelial cells, reducing expression of the integrins e-selectin and vascular cell adhesion molecule 1 (VCAM-1), critical for immune cell migration, in response to TNFα (95). Additionally, a recent transcriptomics study reported upregulation of genes associated with immune evasion in *in vivo* isolated lung *S. mansoni* schistosomula, including genes involved in defence against oxidative stress, potentially minimizing inflammatory effects of reactive oxygen species (ROS) (68). The same study revealed that lung migrating larvae upregulate arginase expression, which can interfere with host nitric oxide (NO) production, thereby suppressing both macrophage and T cell activation (68, 96). Finally, lung stage *S. mansoni* schistosomula, in comparison to maturing larvae isolated at day 13-21, show increased expression of *kunitz protease inhibitor*, a molecule with putative anti-coagulation and anti-inflammatory ability that is also expressed in the adult worm tegument (68, 97). Further work is required to establish the location of these proteins within the schistosomula, with those expressed on the tegument, in contact with the host environment, of particular interest in relation to immune evasion.

Intriguingly, regulatory T cell (Treg) expansion does not appear to occur during schistosomula lung migration (32). This is in comparison to larval stages of nematode infections, where Tregs are induced to modulate the host’s immune system, aiding parasite survival (98–101). It is possible that immune modulation against schistosome larvae is instead mediated by the regulatory cytokine IL-10, which is increased in thoracic lymph node Th2 cells during larval migration of *S. mansoni* (32). Mechanistic experiments, for instance depleting IL-10 or Tregs, are required to directly address if they play any immunoregulatory role during *S. mansoni* larval lung migration.

### Lung Stage Schistosomes as a Vaccine Target

The hope that immunity to schistosomes is possible is supported by the observation that intensity of infection decreases with age, suggesting a build-up of at least partial immunity over time (102). Moreover, correlates of natural immunity have been observed in humans, including type-2 immune factors such as IgE and eosinophils (103–105). It is possible such factors may mediate schistosome killing during their transit through the lung. Indeed IgE responses may be specific to antigens released from dying worms that may cross-react with antigens of invading larvae (106). However, induction of IgE based immunity is not an advisable route for vaccine induced protection, due to the risk of initiating generalised allergic responses, as has been observed in hookworm vaccine trials (107). Alternative strategies to induce protective immunity are therefore required.

Lung migrating larvae have been shown to be the target for protective immune response in mice vaccinated with radiation attenuated (RA) cercariae (14). Radiation treatment decreases larval motility whilst enhancing immunogenicity, allowing the host immune system to mount an immune response that is protective against subsequent infection (108, 109). In non-vaccinated mice parasite survival is around 30%, whereas in vaccinated mice parasite survival to adulthood can be as little as 4%, depending on a number of factors, including the number or vaccinations as well as radiation dose (110, 111). One study utilising a large infectious dose (450 cercariae), found that immune foci surrounding migrating larvae, in comparison to non-vaccinated mice, are larger and observed around both intra-vascular and intra-alveolar parasites, and are thought to block migration (14, 112, 113). Blocking migration may be a particularly effective strategy to inhibit parasite development, bypassing the innate ability of schistosomula to evade immune killing (37, 114).

Much of our understanding of protective immunity against lung migrating schistosomula has come from studies that undertake challenge re-infection post vaccination with RA cercariae (115). A number of non-redundant mechanisms are thought to be required for protection, including CD4^+^ T cell immunity, specifically Th1 cell secretion of IFNγ, pulmonary macrophage activation and NO production, which can result in up to 70% protection against challenge re-infection (116–119). However, it appears that it isn’t as simple as Th1 responses being most effective for vaccine protection, as repeat exposure to RA cercariae induces a Th2 dominated response, and promotes higher levels of protection (>80%) than the Th1-promoting single exposure (120, 121). Further, mice deficient in the IL-4 receptor subunit alpha (IL-4Rα) that develop elevated Th1 responses and impaired Th2 immunity, display reduced vaccine protection, likely due to a reduction in IgG_1_ antibody production (122). Together, this reflects the likelihood that both Th1 and Th2 responses are required for optimal protection in the RA vaccine model, further supported by the fact that protection is enhanced in IL-10 deficient mice, which exhibit elevated Th1 and Th2 responses (123). Mechanistically, what dictates Th1 versus Th2 immunity to RA parasites is not fully understood, but likely depends upon the influence of vaccination dose and frequency on the balance of the immune response that develops (120). Taken together, although the constituents of a protective immune response are still controversial, a robust, mixed Th2/Th1 response may be optimal.

The success of the RA vaccine has led to investigation of lung stage specific antigens in vaccine development (36, 37). Recent work has focused on targeting antibody-accessible schistosomula excretory secretory products, in combination with a Th2 adjuvant (IL-25 or IL-33), with around 70% protection elicited in murine models (124). However, this study had a very short timescale (14 days) between vaccination and challenge with vaccine candidates and so should be treated with caution, as an active immune response to the vaccine would likely still be present at time of challenge. Extending intervals between vaccination and challenge, as well as utilising models with more human-like lung capillary networks, such as non-human primates, are suggestions that have been made to improve the relevance of future tests of lung-stage antigens (111).

### Lung Symptoms in Chronic Schistosomiasis

Pulmonary symptoms can also occur in individuals with chronic schistosomiasis, including lung lesions (observed in chest radiographs), and pulmonary hypertension (22, 23). Pulmonary hypertension is a potentially fatal complication of hepatosplenic disease, occurring in 6.3-13.5% of patients (21, 22). Strikingly, the five year survival rate of untreated individuals with schistosome associated pulmonary arterial hypertension is 69.2% (38). Liver fibrosis due to egg deposition causes portal hypertension that can directly cause pulmonary hypertension (18). Additionally, portal hypertension may lead to the formation of porto-systemic venous shunts, allowing eggs to pass to the lungs, observed both in mice (19) and humans (20). Eggs, or even adult worms, that translocate through these shunts to the lung may obstruct the pulmonary vasculature, either directly or as a result of the host inflammatory response (17, 125). These are thought to be responsible for lung lesions observed *via* chest radiograph, and have been found by histological examination of lung biopsies (23). The clinical importance of pulmonary symptoms in chronic schistosomiasis necessitates increased understanding of the underlying immune responses.

### Lung Immunology of Chronic Schistosomiasis in Murine Models

Murine models of chronic pulmonary schistosomiasis have been developed, in order to better understand the condition and guide future treatments (33). Early studies used surgical ligation of the portal vein to create a shunt from the portal system to the lungs, in order to increase egg translocation to the lungs (126). Utilising this model, granulomas, macrophage and eosinophil rich structures enclosing the eggs, were found in the lungs 10-25 weeks after 50 cercariae *S. mansoni* infection (127). However, these granulomas remained similar in size and cellular constituents over the 10-25 week timescale (127). Immunological modulation (especially decreased granuloma size) is a characteristic feature of liver granulomas (128), and its absence in the surgical ligation model may therefore be a fundamental limitation.

It is possible to model pulmonary effects of chronic schistosomiasis without portal vein ligation. Substantial lung egg deposition peaks at week 17 in mice infected with 30 cercariae, with an average of 189 eggs per lung lobe (33). Notably, this is still vastly reduced in comparison to liver egg deposition, which contains over a million eggs at this timepoint (33). Lung inflammation was observed as an increase in immune cells (especially CD68^+^ macrophages) in granulomas and in perivascular regions (33). The degree of pulmonary vascular remodelling was associated with lung egg burden, supporting the hypothesis that egg deposition contributes to vascular changes seen in human schistosome induced pulmonary hypertension (22, 33). In chronically infected mice, at week 17 post infection, there was a significant positive correlation between numbers of lung eggs and expression of the Th2 cytokine IL-13, suggesting eggs are driving a pulmonary type-2 response (34). The type-2 cytokine IL-4 also increased in the lung in week 17 infected mice, in combination with the type-1 associated cytokines IL-12p70 and TNFα (34). How these different arms of the immune response contribute to the development of pulmonary hypertension in this model is still to be determined.

Pharmacological and genetic intervention studies have shown the importance of immunological processes in regulating pulmonary egg deposition in chronic schistosomiasis. In B cell deficient mice, or mice treated with IL-10R blockade, increased pulmonary egg deposition was observed at 16 weeks post infection, associated with extensive lung cellular infiltration (129). This indicates that immunoregulation mediated by IL-10 or B cells may reduce egg translocation to the lungs. In addition to this, mice that have impaired Th2 development, due to transgenic deletion of the costimulatory molecule CD154, display early egg translocation to the lung during *S. mansoni* infection, before week 8 (130). In this study, CD154^-/-^ mice also displayed reduced iNOS expression, indicating that alterations in vascular mediators such as NO may underlie the increase in pulmonary egg deposition that was observed (130).

Increased definition of the impact of other infection associated immune mediators on the vasculature may be critical to help understand the importance of B cells, IL-10 and Th2 immunity in regulating pulmonary egg deposition. A central role for the vasculature in these processes was reinforced by a recent study, in which schistosome infected mice with heterozygous deletion of *Bmrp2* (bone morphogenetic protein type-II receptor, BMPR-II), the most common genetic cause of pulmonary arterial hypertension (PAH), had enhanced pulmonary egg deposition. In this model, dilated hepatic veins and sinusoids in heterozygous deleted BMPR-II mice increased the ease of portal shunting, leading to elevated pulmonary egg deposition and lung IL-13 cytokine levels (34). Such data strongly indicate that development of novel treatments for schistosome induced pulmonary hypertension will require increased understanding of the critical mediators involved in the interplay between egg-driven inflammation and the vascular system.

The pulmonary immune response to *S. mansoni* eggs can also be modelled by experimental egg injection, where mice are injected intravenously (*i.v.*) with live or cryopreserved eggs, normally following pre-sensitization with eggs injected intraperitoneally (*i.p.*). In this model, following *i.v.* injection, schistosome eggs become lodged in the lungs where they initiate formation of granulomas and production of Th2-biased cytokines (131, 132). This reproducible model for Th2-driven lung inflammation, far quicker than the 16 week+ chronic models of infection, has greatly increased our understanding of Th2-mediated lung granuloma responses (118, 133–135). During chronic infection, pulmonary immune responses to schistosome eggs occur in the context of a pre-existing immune response to larval, adult and egg antigens, which cannot be replicated by experimental egg injection alone. However, comparison of both models may provide substantial insight into the regulation of type-2 immune responses by natural infection, potentially translating to therapeutic benefits in chronic schistosomiasis (136).

### Schistosomiasis and Impact on Allergic Lung Inflammation

Finally, it has been shown that chronic schistosomiasis may regulate pulmonary inflammation during other conditions, such as allergy. Atopic diseases common in high income countries are relatively reduced in lower income countries that have the highest intensity of schistosome infections (1, 137). This inverse correlation between atopy, measured by skin prick test, and schistosome infection in endemic areas (138) has led to suggestions that schistosomiasis protects against lung allergic inflammation. The immunomodulatory properties of helminth infection have been extensively reviewed (139, 140). Murine models have shown chronic *S. mansoni* infection induces regulatory mechanisms that suppress allergic airway responses to model allergens (141). IL-10 producing regulatory B cells (Bregs) are proposed to be key mediators of immune regulation in chronic *S. mansoni* infection, with transfer of Bregs from schistosome infected mice sufficient to reduce allergic airway inflammation (31, 142–144). Expansion of IL-10 producing B cells has also been observed in human *S. haematobium* infection, reduced after praziquantel treatment (31). Notably, the influence of migratory larval stages of schistosomula on allergic conditions is not yet known.

This aspect of infection may be critical to consider in endemic areas, where regular exposure to infected water may make lung migrating schistosomula key influencers of the pulmonary immune responses.

## Conclusions and Future Outlook

Despite the clear pathological consequences of schistosome infection on the lungs, there has been a relative scarcity of refined research into this area in recent years. In both acute and chronic phases, schistosomiasis has debilitating and even fatal pulmonary consequences (7, 22, 52). Immune responses are thought to be responsible for many of the symptoms of pulmonary schistosomiasis, for example the correlation of immune complexes to cough, dyspnoea and interstitial infiltrates observed in chest radiographs in acute schistosomiasis (64). Nonetheless, despite the groundwork being laid by formative older studies of pulmonary schistosomiasis, both in mouse and human (14, 17, 64), our knowledge on this subject is not up to date with the sophisticated and detailed view of the immune system held today.

Despite the many outstanding questions that remain regarding pulmonary schistosomiasis – see **Box 1** – several recent studies that have increased our understanding in this area. For example, the older dogma of acute schistosomiasis as a Th1 dominated response is being questioned by more recent research showing early Th2 cytokine responses in thoracic lymph nodes (32). A focus on T cell responses, often systemic, has meant that there is a notably limited understanding of innate immune responses in lung tissue in both acute and chronic schistosomiasis. In terms of our understanding of human immune responses, controlled human schistosome infection studies have an enormous potential to reveal acute stage pulmonary responses (56). Understanding these responses will be critical to direct the development of novel vaccines or therapeutics for schistosomiasis, as well as the numerous other parasitic infections causing pulmonary pathology (51). Moreover, improved mechanistic understanding of how schistosomes promote or regulate pulmonary immunopathology could also help with rational design of future therapies against diverse lung diseases.

**Box 1: Outstanding questions**

- Which specific aspect of infection (lung migrating larvae, maturing larvae, egg deposition) account for the varied symptoms that can be seen during acute schistosomiasis (e.g. cough, fever)?
- What are the precise kinetics of schistosome lung migration in humans? Increased understanding could be gained by combining experimental human infection studies with research into stage-specific antigens.
- How common is symptomatic acute schistosomiasis in endemic populations? Symptomatic acute schistosomiasis may be under-reported in endemic areas, an issue that could be clarified by more studies of initial exposure in childhood.
- What is the cause of radiographically observed pulmonary lesions in acute schistosomiasis?
- How is the switch between acute and chronic schistosomiasis defined immunologically and symptomatically?
- What are the immunological hallmarks of pulmonary schistosomiasis? Studies in this area have not kept up with either conceptual or technical developments in cellular immunology. For example pulmonary dendritic cell and macrophage cell subsets are thought to be critical for initiation and regulation of immunity, yet which promote or regulate tissue damage against lung stage infection?
- What constitutes a protective immune response in schistosomiasis, and how might we induce this in vaccine studies? Are lung migrating schistosomula suitable targets for vaccine responses?
- How might the immunoregulatory strategies of lung migrating schistosomula be overcome for vaccine or therapy development? On the other hand, might these mechanisms be harnessed to calm over-exaggerated immune responses in inflammatory pathologies?
- Are immunological responses centrally involved in the development of pulmonary hypertension in chronic schistosomiasis? If so, how might this be manipulated therapeutically?

## Author Contributions

ELH wrote the manuscript. AC drafted the figure. AHC, PCC, and ASM critically reviewed and modified the manuscript. All authors contributed to the article and approved the submitted version.

## Funding

This work was supported by a BBSRC CASE studentship (with GSK) to ELH (BB/P504543/1), MCCIR core funding to ASM, and a Royal Society and Wellcome Trust Sir Henry Dale Fellowship (218550/Z/19/Z) and Medical Research Council Centre for Medical Mycology and the University of Exeter (MR/N006364/2) funding to PCC.

## Conflict of Interest

The Manchester Collaborative Centre for Inflammation Research (MCCIR) is a joint venture between the University of Manchester and GlaxoSmithKline (GSK).

The authors declare that the research was conducted in the absence of any commercial or financial relationships that could be construed as a potential conflict of interest.
